# Hamstring Injury Mechanisms and Eccentric Training-Induced Muscle Adaptations: Current Insights and Future Directions

**DOI:** 10.1007/s40279-025-02291-6

**Published:** 2025-08-26

**Authors:** Max H. Andrews, Anthony J. Shield, Glen A. Lichtwark, Patricio A. Pincheira

**Affiliations:** 1 School of Human Movement and Nutrition Sciences, The University of Queensland, Brisbane, QLD 4072 Australia; 2 School of Exercise and Nutrition Sciences, Queensland University of Technology, Brisbane, QLD 4000 Australia; 3 School of Health and Medical Sciences, The University of Southern Queensland, Ipswich, QLD 4305 Australia

## Abstract

Hamstring injuries are a major concern in sports owing to their high incidence and recurrence rates, highlighting the need for a deeper understanding of their mechanisms and prevention. This narrative review aims to inform hamstring injury prevention strategies by examining: (1) the causes of hamstring injuries, (2) the effectiveness of eccentric training in reducing injury risk, and (3) muscle adaptations from eccentric training that may offer protective effects. Hamstring injuries often occur during the late swing phase of running, potentially due to insufficient or delayed neural activation or an inability to generate the necessary force to decelerate the leg and resist active overstretching. In this phase, the hamstrings must produce large eccentric forces while operating at long lengths, placing them in a vulnerable position. Despite the potential of eccentric training to induce muscle adaptations that may reduce injury risk, current research has overly focused on architectural changes, particularly resting fascicle lengthening, without adequately exploring how these adaptations influence the functional behavior of hamstrings during exercise. In addition, the lack of research into adaptations of non-contractile and neural elements in the hamstrings following eccentric training represents a significant gap in the literature. This review argues for a broader focus on these underexplored areas to enhance hamstring injury prevention strategies. Further research is essential to fully understand the mechanisms behind muscle fascicle lengthening after eccentric training. Exploring functional and regional differences in hamstring adaptations and delving deeper into non-contractile and neural elements could enhance injury prevention strategies, potentially reducing the incidence of hamstring injuries.

## Key Points

**Table:** 

Hamstring injuries often occur during the late swing phase of running due to delayed or insufficient neural activation and the high eccentric forces required at long muscle lengths.
While eccentric training is known to increase resting muscle fascicle length, current research overlooks how these adaptations influence hamstring function during exercise.
Greater focus on non-contractile (e.g., titin stiffness, tendon compliance) and neural adaptations (e.g., motor unit recruitment, inhibition) is needed to improve hamstring injury prevention strategies.

## Introduction

Hamstring injuries are prevalent in running sports, contributing to approximately 10% of all injuries in field-based sports [1], with recurrence rates ranging from 15 to 70% [2–6]. Hamstring injuries impact athlete performance and team success [7, 8], which has physical and financial consequences [9]. Despite decades of research and the implementation of strategies, such as resistance training, aimed at improving hamstring strength to mitigate injury risk, the prevalence of hamstring injuries has shown minimal change [10, 11]. This persistent issue highlights the need for a deeper understanding of the causes of hamstring injuries and the development of more effective prevention and rehabilitation strategies.

Despite the high prevalence of hamstring injuries, there is limited consensus on their causes or the factors that might mitigate injury risk [12]. A comprehensive understanding of the underlying causes of these injuries is essential for developing effective prevention strategies. However, compared with the causes of injury, even less is known about the specific muscle adaptations to training that may provide protective benefits. Understanding these adaptations, particularly in response to interventions such as eccentric training, is crucial for optimizing injury prevention programs. By integrating insights from both injury mechanisms and training adaptations, more effective training strategies can be designed to reduce the incidence of hamstring injuries.

Eccentric training programs that increase knee flexor strength effectively reduce the risk of hamstring strain injuries, especially when adherence to the program is high [13–15]. Although eccentric training appears effective in reducing hamstring injury risk, the underlying protective mechanisms remain unclear, as current prevention strategies [16–19] are largely premised on early animal studies conducted decades ago [20–22]. Whether this empirical evidence is sufficient to base hamstring injury and rehabilitation training programs on is questionable. Thus, there is a need to re-examine these theories in light of new evidence and recent technological advances.

This narrative review synthesizes current literature to address three predefined research questions: (1) What are the causes of hamstring injuries, including mechanisms during high-speed running and established risk factors? (2) Is eccentric training effective in preventing hamstring injuries? (3) How do muscles adapt to eccentric training across contractile, non-contractile, and neural domains? These questions shaped the scope of the review and informed the selection of literature.

## Methods

Relevant literature for this narrative review was identified through systematic searches of PubMed and SPORTDiscus, covering studies published up to and including May 2025. Common search terms used across all searches included “hamstring,” “biceps femoris,” “semitendinosus,” and “semimembranosus.” For injury mechanisms and risk factors, these terms were combined with “injury mechanism,” “strain,” “sprinting,” “high-speed running,” “muscle activation,” “muscle tendon unit,” “EMG,” and “risk factors.” To identify studies on the effectiveness of eccentric training in injury prevention, terms included “eccentric training,” “Nordic hamstring exercise,” “injury prevention,” and “injury risk.” For studies on eccentric training-induced adaptations, search terms included “eccentric training,” “fascicle length,” “sarcomere,” “strength,” “extracellular matrix,” “titin,” “tendon,” “muscle function,” “motor unit,” and “neural adaptation.” Titles and abstracts were screened for relevance, and full-text articles were retrieved when aligned with the research questions. Reference lists of included articles were also reviewed to identify additional sources.

## Causes of Hamstring Injuries: A Prelude to Prevention Strategies

### Mechanisms of Hamstring Injury in High-Speed Running

**Fig. 1:**
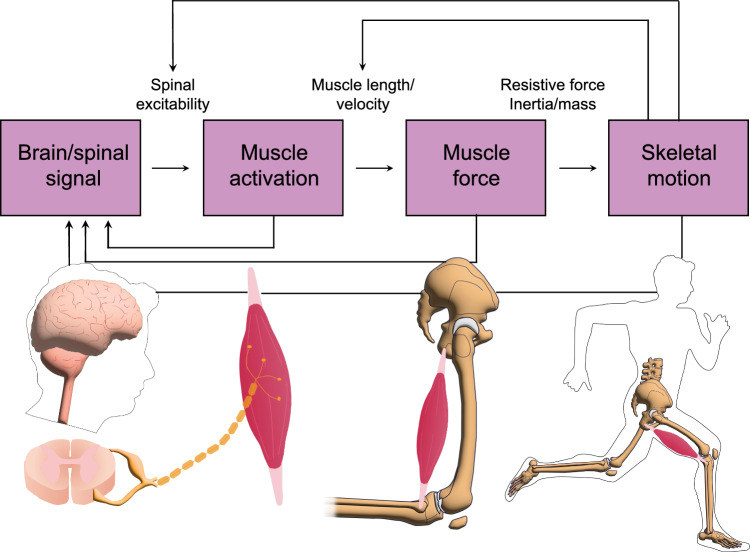
Integration of neural, muscular, and skeletal systems to produce running

Over 80% of hamstring injuries occur during high-speed running, predominantly affecting the biceps femoris long head (BFlh) muscle [23, 24]. As such, this review focuses specifically on running-related hamstring injuries. Despite extensive research, conflicting views persist regarding the precise etiology of these injuries [25]. High-speed running requires precise coordination of multiple elements of the neuromusculoskeletal system (Fig. 1). Disruptions at any point in this coordination may lead to hamstring injuries. Evidence indicates that running-related hamstring injuries typically occur during two distinct phases: opposing external forces during early stance [23] or active lengthening in the late swing phase [26].

#### Hamstring Injury Risk in Early Stance

The early stance phase of running has been argued to be a potential point for hamstring injury [27, 28]. During this phase, the neural system activates the hamstrings prior to foot contact to generate the necessary hip extension and knee flexion torques. These torques are required to produce the ground reaction forces needed for decelerating the shank [29]. The high forces, and hence, high stresses (i.e., force per unit cross-sectional area) may expose the hamstrings to injury [12, 30]. Another potential contributing factor is anterior pelvic tilt, which may increase hamstring strain by amplifying both active lengthening and passive tension demands during stance [31–33], though evidence supporting this link remains limited. Despite the substantial loads, hamstring muscles do not appear to undergo lengthening contractions during early stance in running [26, 34]. Moreover, the muscle lengths in the early stance phase of running are well within their normal operating range [35], which suggests the hamstring muscle fibers are unlikely to be stretched considerably in the early stance phase of running. However, it is possible that incorrect force generation or delayed activation could result in unexpected hamstring lengthening and increase the risk of injury [36, 37].

#### Hamstring Injury Risk in Late Swing

The hamstrings play an important role in terminating the swing phase of running, where they must sufficiently generate force to decelerate the leg [37]. This process requires precise control of the neuromusculoskeletal system to prevent injury from increased strain or rapid force increases. During late swing, as the hip reaches peak flexion and the shank accelerates forward, the hamstrings act eccentrically, generating large hip extension and knee flexion moments [12, 29]. This places the hamstrings in a vulnerable position, requiring them to produce large eccentric forces to resist stretching at long lengths. Animal studies suggest that muscle injuries are most often incurred when exposed to high strains at long muscle lengths [38–40]. High activation is necessary to generate the required force to resist this overstretching, but if it fails to adequately resist muscle stretching, even a small strain can cause injury [41]. Therefore, hamstring injuries during late swing in running may result from insufficient or delayed neural activation, or an inability to produce sufficient force to decelerate the leg and resist active overstretching.

Maximal activation of the hamstring muscles appears to coincide with peak muscle–tendon unit (MTU) stretch during running [26, 34, 42, 43]. During late swing in running, the hamstring MTUs are at significantly longer lengths compared with the rest of the gait cycle [44, 45]. Musculoskeletal modeling of running suggests that BFlh MTU length peaks at 112% of upright standing length—2–3% more than semimembranosus and semitendinosus [34]. This places significantly more strain on the BFlh MTU [26, 34]. As running speed increases, the hamstrings are activated to a greater extent [46, 47], and during accelerative running, they are stretched to longer lengths and at faster lengthening velocities [48]. Animal experiments suggest that muscle injury is influenced not only by the magnitude of strain but also by the combination of strain and activation [38]. Given the maximal activation and extensive lengthening of the BFlh in the late swing phase of running, it may be more susceptible to strain injuries compared with other hamstring muscles [26, 34, 42, 43].

### Risk Factors for Hamstring Injuries

Elevated muscle fiber stress at extended lengths, while the muscle is stretching, likely contributes to hamstring injuries. As established above in Sect. 3.1, these factors can be influenced by eccentric strength (the ability to generate and resist high forces) and fascicle length (which affects net strain).

#### Eccentric Strength

Greater eccentric strength is believed to help reduce injury risk by generating more force to oppose excessive strain on the hamstrings [49]. However, evidence regarding the predictive value of eccentric hamstring strength for injury risk is mixed. Some studies have indicated that athletes with lower eccentric hamstring strength are at greater risk of hamstring injury [37]. For example, Australian Rules football and soccer players with pre-season eccentric strength below 279 N and 337 N, respectively, had a greater than fourfold increased risk of injury in the subsequent season [24, 50]. Moreover, the risk of hamstring injury decreased by about 9% for every 10 N increase in eccentric knee flexor strength [24]. Conversely, other research, including a meta-analysis, has not found a significant association between eccentric strength and future injury risk [51–53], which may be attributed to several limitations commonly present in these studies. These limitations often include small sample sizes that impede the detection of small-to-moderate associations, a lack of multiple measurements throughout the season, and an absence of player exposure data [54]. Despite these inconsistencies, eccentric strength remains an important risk factor to consider for injury prevention because it may play a role in the ability of the hamstring muscles to withstand excessive muscle strain during active lengthening [55].

#### Muscle Fascicle Length

A large prospective study found that athletes with resting fascicle lengths shorter than 10.56 cm are 4.1 times more likely to suffer a hamstring strain [24]. Athletes with a history of hamstring injury typically have shorter resting fascicle lengths [56], which shifts the optimum length (relative to the force–length relationship) to shorter lengths [57]. In contrast, eccentric training has been shown to increase resting fascicle length [17, 18, 58, 59], with every 0.5 cm increase associated with a 21% reduction in hamstring injury [24]. While this reduction in injury risk is thought to result from decreased fiber strain due to serial sarcomerogenesis [16–19], there is currently limited human muscle data to support this assertion [60, 61]. Consequently, the relationship between fascicle length, sarcomere adaptations, and the resultant effect on injury risk remains largely theoretical. Evidence of the roles of muscle adaptations to eccentric training and the potential effect on injury are discussed in detail below.

## Preventing Injury: How Muscles Adapt to Eccentric Training

Eccentric training interventions, particularly those incorporating the Nordic hamstring exercise (NHE), demonstrate evidence for reducing hamstring strain injury risk [62–65]. Meta-analytic evidence indicates that programs including the NHE reduce injury incidence by approximately 51% [66, 67]. Although one meta-analysis did not observe a statistically significant effect [13], this finding appears attributable to poor adherence across several included studies [64, 68, 69]. When analyses are restricted to compliant participants, eccentric training demonstrates substantial protective effects, with injury risk reductions approaching 65% [13]. The weight of evidence supports including eccentric training as a primary strategy in hamstring injury prevention, provided that the program is adhered to.

The remainder of this section examines how eccentric training may confer this protective effect by exploring adaptations in the hamstring muscles. This section synthesizes current evidence on these adaptations, focusing on eccentric training in human hamstrings. Where direct evidence is lacking, relevant findings from animal models, other muscle groups, or general resistance training are included. Most of the research on eccentric training of the hamstrings has focused on contractile adaptations, but there has been comparatively little investigation into changes in non-contractile tissues or neural control in the hamstrings. Investigating these underexplored areas could shed light on how specific muscle adaptations from eccentric training contribute to reducing hamstring injury risk.

### Contractile Tissue Adaptations

#### Adaptation of Fascicle Length

A key target for training is to improve tolerance to active muscle fiber stretch, particularly during high tension when strain injuries are more likely [41]. Protective adaptations are likely induced by training that combines high muscle force and strain, a characteristic of eccentric training [70, 71]. Early weeks of eccentric training typically induce rapid increases in resting hamstring fascicle length [17, 18, 59, 61]. Subsequent training leads to gradual increases in resting fascicle length over extended periods [17, 72, 73]. However, it remains unknown if this increase in resting fascicle length reduces the strain experienced by muscles during lengthening (i.e., operating lengths).

**Fig. 2:**
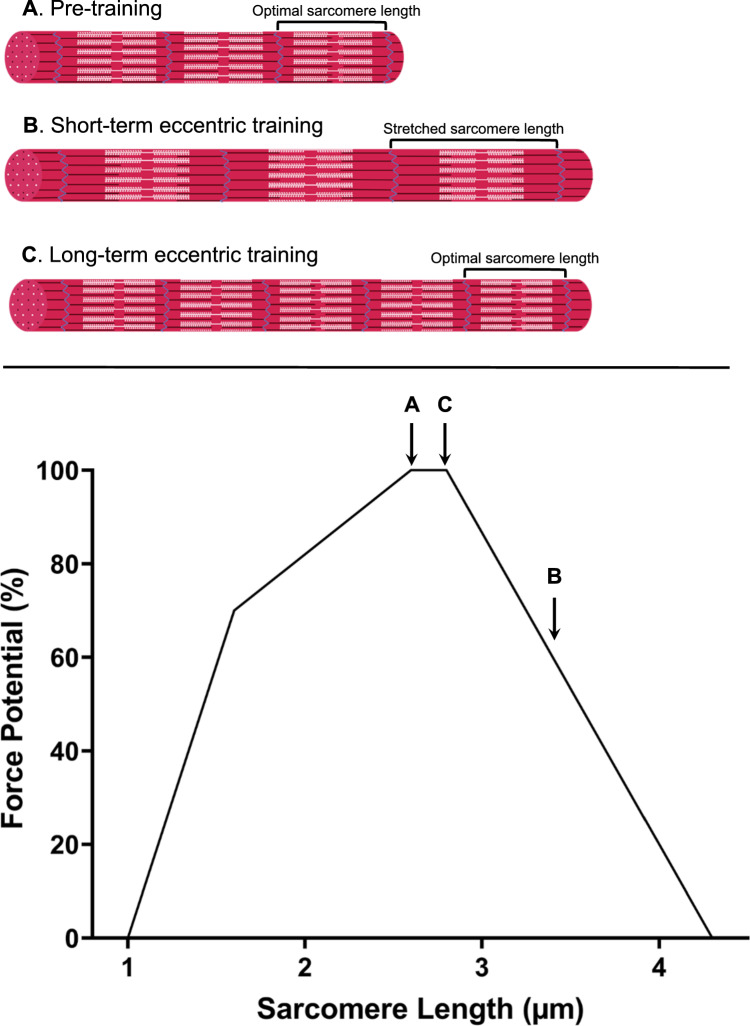
Theoretical relationship between sarcomere length and force-generating potential as a function of eccentric training: **A** Sarcomeres at their optimal length (~ 2.64 μm) before undertaking eccentric training, yielding maximum force potential (100%); **B** sarcomeres in series stretched to ~ 3.4 μm, reducing force potential (~ 50%) due to suboptimal filament overlap, representing early eccentric training adaptation; and **C** increased number of sarcomeres in series at or near optimal lengths, restoring high force potential (~ 100%) and illustrating the long-term adaptation of sarcomerogenesis after eccentric training. This figure illustrates the progression from initial sarcomere elongation to increased serial sarcomere number

While eccentric training has the potential to increase resting fascicle length in the BFlh muscle [18, 19], the precise mechanisms underpinning this have not been fully established. Eccentric training is postulated to stimulate an increase in fiber length through either an increase in serial sarcomere number or elongation of individual sarcomeres [74]. These two adaptations would have different effects on the risk of hamstring muscle strain injury (Fig. 2). Eccentric training forces the muscle to operate in an elongated state [75], which may stretch sarcomeres beyond their optimal length [76]. Sarcomeres have a reduced capacity to produce force outside their optimum length because they have less actin-myosin overlap at short or long lengths [77]. As such, sarcomere length appears to be highly regulated, so that sarcomeres operate within their optimum length range [78]. According to the sarcomerogenesis hypothesis, muscles adapt to maintain optimal sarcomere lengths for functional tasks undertaken [79, 80]. When muscles are required to produce force at longer lengths, fascicles presumably adapt by increasing the number of sarcomeres in series, reducing the stretch experienced by individual sarcomeres at these longer lengths [76, 79–81]. This adaptive response highlights that sarcomerogenesis is seemingly driven by the need to optimize sarcomere length within the force–length relationship [82].

Of the potential adaptations to eccentric exercise that might enhance hamstring resistance to strain injury, sarcomerogenesis has received the most attention. Only recently has it been possible to understand how sarcomeres adapt to eccentric hamstring exercise, using a technique known as microendoscopy to directly measure sarcomere length [83, 84]. Initial increases in fascicle length from early (3 weeks) eccentric training stem mainly from increased resting sarcomere length rather than serial sarcomere number [61]. After 3 weeks of eccentric training, average sarcomere length increases at rest, which places sarcomeres in an overstretched position with reduced force-generating capacity due to fewer actin–myosin cross-bridges [77].

More recent evidence suggests that after 9 weeks of eccentric training, sarcomeres return to pre-training lengths while fascicles are significantly elongated, suggesting that serial sarcomerogenesis had occurred [60]. This increase in serial sarcomere number likely helps protect against muscle strain injuries by potentially enabling sarcomeres to operate on the ascending limb of the force–length curve, maintaining optimal lengths and avoiding overstretching during eccentric contractions. Thus, serial sarcomere addition appears to reduce the strain on individual sarcomeres and likely enhances the ability of the hamstring muscles to generate force effectively without becoming overstretched. However, the implications for injury prevention remain unclear, as there are no measurements of fascicle or sarcomere behavior during active muscle stretching.

Variable muscle morphology in the hamstrings could contribute to a heterogeneous strain distribution within muscles [85, 86], which in turn may influence adaptive responses. For instance, the BFlh has nonuniform fascicle lengths, with longer fascicles proximally and shorter ones distally [87], accompanied by heterogeneous sarcomere lengths [61]. Computational modeling studies suggest that strain amplitude in the hamstrings during high-speed running is often greatest near the musculotendon junction [88, 89]. However, very little is known about in vivo operating fascicle and sarcomere lengths during exercise, especially considering the biarticular nature of the hamstrings, where differing contributions from the hip and knee joints likely lead to varied fascicle behavior during force generation. Heterogeneous adaptations have been demonstrated in BFlh in response to eccentric training [60, 61]. In a knee-dominant exercise such as the NHE, the longer proximal fibers should experience less strain than the shorter distal fibers if all fibers stretch by a similar absolute amount [90], possibly driving greater adaptive responses observed in the distal region [60, 61]. This inference, however, remains largely untested, highlighting the need for further research to understand the strain distribution across the muscle during eccentric exercises and to explain how these regional differences in strain patterns relate to nonuniform muscle adaptation.

#### Adaptation of Muscle Size and Strength

Increasing hamstring muscle size not only enhances the force-generating capacity [91] but also theoretically reduces muscle fiber stress at a given level of muscle force, as stress is inversely proportional to area. This increased strength and reduction in stress may contribute to the ability of the hamstring muscles to resist overstretching and potentially prevent hamstring strain injuries [16, 49, 72]. Consequently, hypertrophy may play an important role in preventing hamstring strain injuries. Indeed, hypertrophy of hamstrings has been observed within 6–10 weeks of eccentric training [72, 92]. It is important to mention that not all resistance training methods equally stimulate hypertrophy across all hamstring muscles. Several studies have shown that exercises such as the NHE preferentially induce hypertrophy in the semitendinosus rather than the BFlh [72, 93, 94]. A recent study suggests that hypertrophy of the BFlh requires exercises that allow for greater excursions than typically experienced in the NHE [93]. Further evidence has demonstrated nonuniform hypertrophy within the hamstrings, with greater increases in the central regions of the semitendinosus and BFlh following 10 weeks of training, particularly with hip extension exercises [95]. However, this nonuniform hypertrophy does not necessarily indicate that fibers in the central regions are larger, as these differences could be related to anatomical constraints or the ability of the muscle to bulge, accommodating increases in fiber cross-sectional area and length. Strength is also not only a function of muscle structure but also the ability to activate muscle tissue for force generation. The ability of the nervous system to stimulate muscle fibers to generate force is explored in Sect. 4.3.

### Non-contractile Tissue Adaptations

#### Adaptation to Extracellular Matrix

Extracellular matrix (ECM) remodeling may protect muscles such as the hamstrings following resistance training by contributing to passive tension during stretch and reducing strain on muscle fibers [96]. These adaptations occur more rapidly than contractile changes and may contribute to resisting stretch and preventing excessive strain [97]. However, the specific adaptations of the ECM from eccentric training of the hamstrings are poorly understood. Initial ECM changes after eccentric training, observed in animal models and other human muscles (but not yet in the hamstrings), involve de-adhesion, facilitated by tenascin-C, which creates an adaptive environment necessary for remodeling but can temporarily reduce strength [98–101]. These ECM adaptations appear to occur quickly, preceding contractile changes [97]. The de-adhesive phase may increase vulnerability to injury, highlighting the need for a clearer understanding of the ECM adaptation timeline [97]. Later stages of ECM remodeling show increased collagen synthesis [102], potentially contributing to greater stiffness of the ECM and tensile strength. Such non-contractile tissue adaptations might help redistribute stress across the hamstrings, potentially reducing injury risk [103]. Despite the potential role of ECM adaptations in preventing hamstring strain injuries, most current knowledge is speculative and based on research involving other muscles, with limited direct evidence for the hamstrings.

#### Adaptation to Titin

Eccentric training may protect hamstring muscles from strain injuries by increasing titin stiffness [104]. This increased stiffness may reduce the extensibility of sarcomeres during active lengthening and increase force generation by modulating the actin–myosin interaction [105, 106]. Titin stiffness increases more during active lengthening [107] due to Ca^2+^ influx, which appears to promote titin binding to actin [108]. Although speculative, eccentric training might affect titin’s role in regulating sarcomere stiffness and reducing strain injury risk, highlighting the need for further research to confirm these postulations.

#### Adaptation to Tendinous Tissue

During the late swing phase of high-speed running, while the MTU experiences the greatest active lengthening, muscle fibers themselves may undergo minimal length change [109, 110]. The degree of strain experienced by muscle and tendinous tissue depends on the force generated and the stiffness of the tendon and aponeurosis [111]. Aponeurosis geometry may also influence strain magnitudes at the muscle–tendon junction [112], with long-length eccentric training potentially inducing increases in the aponeurosis area [93]. More compliant tendinous tissues could potentially enable muscles to remain closer to their optimal lengths during eccentric contractions [90, 113, 114]. That is, as force increases, the tendinous tissue stretches instead of the muscle fibers. However, the effects of eccentric training on hamstring tendinous tissues are not well understood. Recent work has shown that while eccentric training stimulates hamstring muscle hypertrophy, there are minimal changes to aponeurosis or free tendon geometry [95]. In addition, findings from short-term training studies suggest that changes in tendon modulus largely account for stiffness adaptations [115]. Thus, while tendon compliance may theoretically reduce muscle fiber strain, further research is needed to explore how eccentric training affects muscle and tendinous tissue behavior.

The role of tendinous tissue strain in protecting muscle fibers from excessive strain during lengthening contractions requires more research. Modeling studies of the hamstrings in running have demonstrated discrepancies between MTU and fiber length changes during contractions [89, 116], with fibers operating within a narrower length range and producing force at more optimal lengths [117, 118]. Ultrasound and musculoskeletal modeling have shown that BFlh elastic tendinous tissue stretches more than muscle fibers during slow eccentric contractions [90, 113, 114], supporting previous simulations [89, 116]. This relationship is likely task-dependent, as changes in joint configuration can alter MTU and fascicle length mechanics [90]. While the effects of eccentric training on these dynamics remain unclear, no studies have investigated how the mechanical properties of the hamstring muscle, such as MTU compliance and fascicle stretch, change in response to training. Existing studies have only examined these properties at a single time point [90, 113, 114]. While these cross-sectional studies have shown that the MTU absorbs much of the stretch during eccentric contractions, fascicle stretching occurs predominantly when force is highest [90, 113, 114]. Given the limited understanding of MTU interactions during exercise, further research is needed to fully explore the potential for tendinous tissues to buffer stretch in muscle fibers during eccentric contractions and the implications for hamstring injury prevention.

### Neural Adaptations

#### Increasing Neural Drive

Adaptations to the nervous system that increase the force-generating capacity of muscle might also reduce the risk of hamstring injury. Eccentric training at high intensities appears to induce greater increases in strength compared with concentric training [119, 120], largely because eccentric contractions generate more force for a given level of neural activation [121, 122]. While neural adaptations leading to improved voluntary activation have been reported in several muscles following eccentric training [123, 124], there is limited evidence for these changes in the hamstrings specifically. Investigating these neural adaptations in the hamstrings could provide valuable insights into reducing injury risk and improving performance.

#### Motor Unit Adaptations

Motor unit adaptations following resistance training may further enhance force generation, as strength is influenced by motor unit size and firing frequency [125]. Research on other muscles indicates that, early in resistance training, thresholds for motor unit recruitment appear to decrease [126, 127]. However, these changes seem to revert with continued training, as muscle fiber adaptations become more prominent [128]. While increased firing frequencies have been proposed to improve force production [127], research findings remain inconsistent [129, 130] and no studies have specifically investigated these adaptations in the hamstrings. Recent research suggests that hamstring motor unit behavior is influenced by joint angle and muscle length [131, 132]. However, the complexity of the biarticular nature of the hamstrings poses challenges in assessing their motor unit properties, which likely contributes to the limited research in this area. Understanding these neural adaptations is crucial for improving training protocols to enhance hamstring force production and prevent injuries.

Research should focus on motor unit properties such as recruitment and de-recruitment thresholds, mean discharge rate, discharge rate variability, and motor unit firing–torque relationships [133, 134]. Evidence indicates that neural drive to muscles varies according to functional demands, with lower recruitment thresholds in faster contractions [135] and higher mean discharge rates contributing to increased force production [136]. In addition, analyzing motor unit firing–torque relationships can shed light on the conversion and transmission of neural drive to muscle force [133, 134], which is essential for optimizing performance and reducing injury risk.

#### Inhibitory Neural Control Mechanisms

Eccentric training may reduce inhibitory neural mechanisms, enabling the hamstrings to generate greater force [137]. Protective mechanisms such as the stretch response from muscle spindles or tension-limited inhibition from the Golgi tendon organs [138–140] can protect muscles from excessive stress and strain. However, this inhibition may also limit strength adaptations. Indeed, inhibitory responses appear to be upregulated in previously injured limbs, which may impede rehabilitation progress and elevate reinjury risk [141, 142]. By downregulating these inhibitory responses, eccentric training may increase force generation during lengthening contractions [122, 143]. While untrained individuals show increased muscle activation and force with superimposed nerve stimulation during eccentric contractions, this effect diminishes with training, indicating improved neural recruitment [144, 145]. Nonetheless, direct evidence on whether eccentric training reduces hamstring neural inhibition and if this downregulation protects against injury is lacking, warranting future research.

#### Regional Adaptations to Muscle Activation

The number and size of activated motor units appear to vary between muscles and across regions within a muscle [146, 147]. Evidence suggests that the activation of the hamstring muscles is nonuniform [148–150], which may lead to regional differences in stress and strain distribution. While it is theorized that areas of muscle with greater neural drive can produce more force and may therefore be more resistant to lengthening, these ideas remain speculative. Existing studies are cross-sectional and only provide a snapshot of hamstring activation during exercise without assessing how regional activation changes with training or its impact on strain-based adaptation.

The recruitment patterns of hamstring muscles are influenced by the relative contributions of the hip and knee joints during exercise. For instance, knee-dominant exercises, such as the NHE, typically activate the semitendinosus more than hip-dominant exercises, which tend to activate the hamstrings more uniformly [148, 151–153]. Previous studies have shown that muscle hypertrophy patterns may align with the metabolic activity observed through functional magnetic resonance imaging (fMRI) [72, 151]. fMRI detects the extent of muscle activation following exercise by capturing signal intensity changes related to metabolic activity within the muscle [154].

However, surface electromyography (sEMG) studies have shown inconsistent results regarding preferential recruitment of hamstring muscles under different activities. For example, some studies indicated similar activation levels of semitendinosus and BFlh during knee-dominant exercises [155, 156], while others report higher BFlh activation during knee-dominant and hip-dominant exercises [157, 158]. Despite BFlh often being less activated and experiencing less hypertrophy than other hamstring muscles during the NHE, numerous studies demonstrate significant BFlh activation [157, 159], particularly compared with the eccentric phase of other exercises [151].

Understanding the common techniques for assessing muscle activation is crucial for interpreting the varying results seen across studies. sEMG provides an indirect measure of net motor unit activity by detecting the sum of all action potentials from recruited and active motor units [160], although its lower spatial resolution may contribute to discrepancies in findings. In contrast, high-density EMG (HD-EMG) provides enhanced spatial resolution by recording electrical signals from multiple muscle compartments and can identify individual motor unit firing times using blind source separation, offering deeper insights into muscle activation patterns [133, 134]. However, HD-EMG is currently limited to isometric contractions and cannot estimate motor unit properties during dynamic contractions.

Understanding the within-muscle regional variation in hamstring activation is crucial to identifying the fundamental determinants of stress and strain distribution within the hamstring muscles, as well as how these regions adapt to training. While several studies have reported distinct activation patterns within the hamstring muscles, the methodologies employed warrant critical examination. For instance, the middle region of semitendinosus generally has the highest activity, while the distal region of the BFlh is the most active, especially during the NHE [161]. Knee-dominant exercises such as the NHE lead to lower activity in the distal semitendinosus and higher activity in the middle and proximal regions, whereas hip-dominant exercises such as the stiff-leg deadlift show more uniform activation across regions [148]. These findings may be limited by single columns of electrodes, which restrict the recorded muscle area, whereas studies using larger matrices are recommended to enhance activation mapping resolution [133]. Recent research using larger electrode matrices has demonstrated that distinct neural drives exist between the proximal and distal regions of the semitendinosus during submaximal contractions [132]. These regional differences may arise from variations in muscle architecture and innervation patterns, highlighting the need for further research to examine how these muscles adapt to training. This need is particularly pertinent in light of evidence showing regional differences in the amplitude from sEMG during dynamic movements [148, 161]. sEMG amplitude reflects the sum of muscle fiber action potentials [160], which can vary due to factors such as muscle length changes and electrode placement. However, these amplitude variations do not necessarily indicate regional differences in motor unit behavior, as motor unit action potentials themselves may not directly vary with regional activation patterns. More studies specifically investigating motor unit properties in the hamstrings are needed to fully understand these regional variations and their implications for muscle adaptation.

## Conclusions

**Fig. 3:**
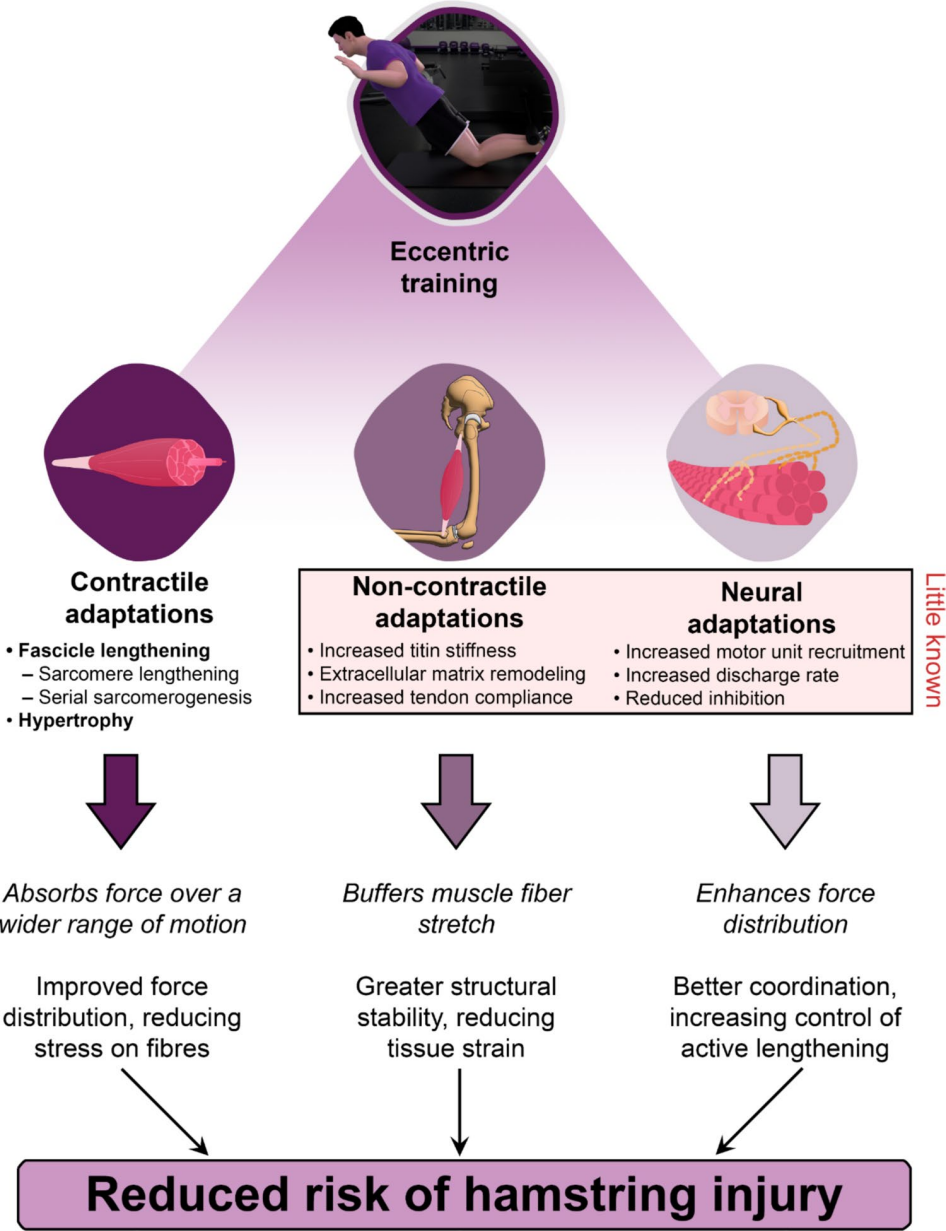
Illustration of potential mechanisms that may underlie the protective adaptations of the hamstrings in response to eccentric training, potentially reducing injury risk. While there is strong evidence for contractile adaptations such as fascicle lengthening and hypertrophy, less is known about non-contractile and neural adaptations in human hamstrings. Non-contractile changes, such as increased titin stiffness, extracellular matrix remodeling, and enhanced tendon compliance, are hypothesized but require further investigation. Neural adaptations, including increased motor unit recruitment, increased motor unit discharge rates, and reduced inhibitory feedback, may also play a role, though evidence for the hamstring muscles is limited

This review examined the complex mechanisms underlying hamstring injuries resulting from high-speed running, which are mainly caused by excessive strain and high muscle activation during running. The BFlh muscle is especially vulnerable owing to its maximal activation and lengthening during the late swing phase of running. Factors such as short resting fascicle lengths and low eccentric strength further reduce the ability of the hamstrings to resist overstretching, yet their behavior during exercise remains poorly understood. Although injury prevention strategies targeting increased muscle fascicle length and eccentric strength show promise, the underlying mechanisms remain unclear. Existing research predominately focuses on passive muscle fascicle lengths rather than operating lengths during contractions. In addition to contractile tissue adaptations, such as changes in muscle fascicle length, this review suggests that adaptations in non-contractile and neural elements may also offer protective benefits (Fig. 3). Exploring these less-studied areas could improve strategies for preventing hamstring injuries.
